# Supplementary material for: Using the Concept of Health Literacy to Understand How People Living with Motor Neurone Disease and Carers Engage in Healthcare: A Longitudinal Qualitative Study
Source: Healthcare (Basel). 2022 Jul 24;10(8):1371. doi: 10.3390/healthcare10081371 (PMC9330690; doi:10.3390/healthcare10081371)
Supplement: Supplementary file 1 [file healthcare-10-01371-s001.zip › healthcare-1791820-supplementary.pdf]

Supplementary Material

Supplementary Table S1 – Interview sample per time point.

Time point	n= plwMND	Face-to-face or telephone interview	Number of plwMND who discontinued	Reason for withdrawal
T1	19	All face-to-face		N/A (baseline)
T2	15	All face-to-face	4	3 = death<br>1 = declined further participation; no reason provided
T3	12	1 telephone	3	3 = death +/- unwell
**Time point**	**n= carers**	**Face-to-face or telephone interview**	**Number of carers who discontinued**	**Reason for withdrawal**
T1	15	1 telephone		N/A (baseline)
T2	12	2 telephone	3	1 = declined further participation; no reason provided<br>2 = bereaved carers*
T3	7	1 telephone	5	1 = declined further participation; citing insufficient time<br>3 = bereaved carers<br>1 = T2 bereaved carer*

* Except for one bereaved carer, carer participants whose family members died or were too unwell to participate were not asked to continue. One carer, bereaved following the T1 interview, agreed to participate in a T2 interview. This carer is subsequently considered to have withdrawn from the study at T3.

Supplementary Table S2 – Interview composition.

Time point	Number of interviews conducted individually	Number of interviews conducted jointly
T1	22	6
T2	14	7
T3	13	3

Supplementary Material: PlwMND Interview Guides

Interview T1

Decision Making

Tell me about a healthcare decision you've had to make (or are about to make).

Who else do you involve in these decisions? How much, or how, do you discuss this elsewhere e.g. at home?

What makes you feel involved, or not involved, in making these decisions?

Have you heard of Advance Care Planning? If so, can you tell me about your experience with ACP?

Communication changes (if applicable)

Tell me how have changes in your speech or communication changed the way you are doing things? (e.g. making phone calls, completing forms, writing cards/letters, typing emails?)

Have changes in your speech changed the way you interact with your health professionals? Or changed decision-making in any way?

Gastrostomy (if applicable)

Tell me about any decisions you have made regarding eating and drinking, and/or a feeding tube?

How has the feeding tube changed things for you?

In what ways has the feeding tube made things easier/better versus more difficult/worse?

Interview closure

Tell me, what do you think is the most challenging thing about decision-making?

Tell me, what do you think helps your decision-making?

Interview T2

Decision-making

Tell me about the decisions you have made since we last met?

Who else is involved in these decisions? How much, or how, do you discuss this elsewhere e.g. at home?

Have you been involved with Advance Care Planning (ACP)? Can you tell me about your experience with ACP?

In what ways were you involved in these decisions? How do you feel involved?

Communication changes

Has your speech or communication changed? How has this changed the way you interact with your health professionals? Has it changed decision-making in any way?

Can you describe a time the changes in your speech or communication had an impact on your involvement in decision-making?

People with MND sometimes report changes in memory or concentration. Is this happening to you? Is it impacting decision-making?

Gastrostomy (if applicable)

Tell me about any decisions you have made regarding eating and drinking and/or a feeding tube.

How has the feeding tube changed things for you?

In what ways has the feeding tube made things easier/better or more difficult/worse.

Wrap up

Anything else you'd like to share?

Interview T3

Decision-making

Tell me about an important decision you've had to make, and how you went about making it.

Did you feel you had enough time? Did you ask questions? Did you feel listened to?

Tell me how making decisions/choices might have changed over time.

Advance care planning is one way we can communicate our decisions. Tell me about your experience with ACP?

Seeking and receiving information

Tell me about information you've received about MND.

Did you ask for information?

Did you seek information from alternative sources?

Can you give an example?

How useful or helpful was it? Did it help you make a decision?

The impact of time

Did the process of making decisions) change with time?

Communication changes

Has your speech or communication changed? How has this changed interactions with HCP

Can you describe a time changes in your speech or communication had an impact on your involvement in your healthcare?

People with MND sometimes report changes in memory or concentration. Is this happening to you? How is it impacting your healthcare or decision-making?

AAC users:

Tell me about using AAC.

Where, with whom do you use it? Do you use AAC in medical appts?

Gastrostomy (if applicable)

Tell me about any decisions regarding eating and drinking and/or feeding tubes.

Have these decisions or thoughts

Supplementary Material: Carer Interview Guides

Interview T1

Decision Making

Tell me about a healthcare decision that you or [NAME] have had to make (or are about to make).

Who else do you involve in these decisions? How much, or how, do you discuss this elsewhere e.g. at home?

What makes you feel involved, or not involved, in making these decisions?

Have you heard of Advance Care Planning? If so, can you tell me about your and [NAME] experience with ACP?

Communication changes (if applicable)

Tell me how have changes in [NAME] speech or communication changed the way you are doing things at home or in clinic? (e.g. making phone calls, completing forms, writing cards/letters, typing emails?)

Have changes in [NAME] speech changed the way you or [NAME] interact with your health professionals? Has it changed decision-making in any way?

Gastrostomy (if applicable)

Tell me about any decisions you or [NAME] have made regarding eating and drinking, and/or a feeding tube?

How has the feeding tube changed things for you? In what ways has the feeding tube made things easier or worse?

Interview closure

Tell me, what do you think is the most challenging thing about decision-making?

Tell me, what do you think helps your decision-making?

Interview T2

Decision-making

Tell me about the decisions you or [NAME] have made since we last met?

Who else do you involve in decisions? Tell me about how you make decisions e.g. do you or [NAME] discuss this elsewhere?

In what ways are you involved in decisions?

Have you been involved with Advance Care Planning (ACP) since we last met?

Communication changes

Has [NAME] speech or communication changed? How has this changed interactions with health professionals? Has it changed decision-making in any way?

How have things changed for you now that [NAME] speech/comm has changed? What do you / family do differently?

PwMND sometimes report changes in memory or behaviour. Have you noticed this happening to [NAME]? Is it impacting decision-making?

Gastrostomy (if applicable)

Tell me about any decisions you or [NAME] have made regarding eating and drinking and/or a feeding tube.

How has the feeding tube changed things for you? In what ways has the feeding tube made things easier or worse?

Wrap up

Anything else you'd like to share?

Interview T3

Decision-making

Tell me about an important decision you or [NAME] had to make, and how you went about making it.

Did you feel you had enough time? Did you ask questions? Did you feel listened to?

Tell me how making decisions/choices might have changed over time.

Advance care planning is one way we can communicate our decisions. Tell me about your and [NAME] experience with ACP?

Seeking and receiving information

Tell me about information you've received about MND.

Did you ask for information? What were you given? Did you seek information from alternative sources? Can you give an example?

How useful or helpful was it? Did it help you or [NAME] make decisions?

The impact of time

Did the process of making decisions change with time?

Communication changes

How have changes in [NAME] speech/comm impacted you? How has this changed interactions with healthcare professionals?

Can you describe a time changes in [NAME] speech/comm had an impact on your or [NAME] involvement in healthcare?

PwMND sometimes report changes in memory. Is this happening to [NAME]? How is it impacting healthcare or decision-making?

AAC users:

Tell me about [NAME] use of AAC. Where, with whom does use it? (home, public, medical appts? etc)

Gastrostomy (if applicable)

Tell me about any decisions regarding eating and drinking and/or feeding tubes. Have these decisions or thoughts changed over time?

Supplementary Material

Supplementary Table S3. Participant longitudinal functional assessment scores (ALS Functional Rating Scale).

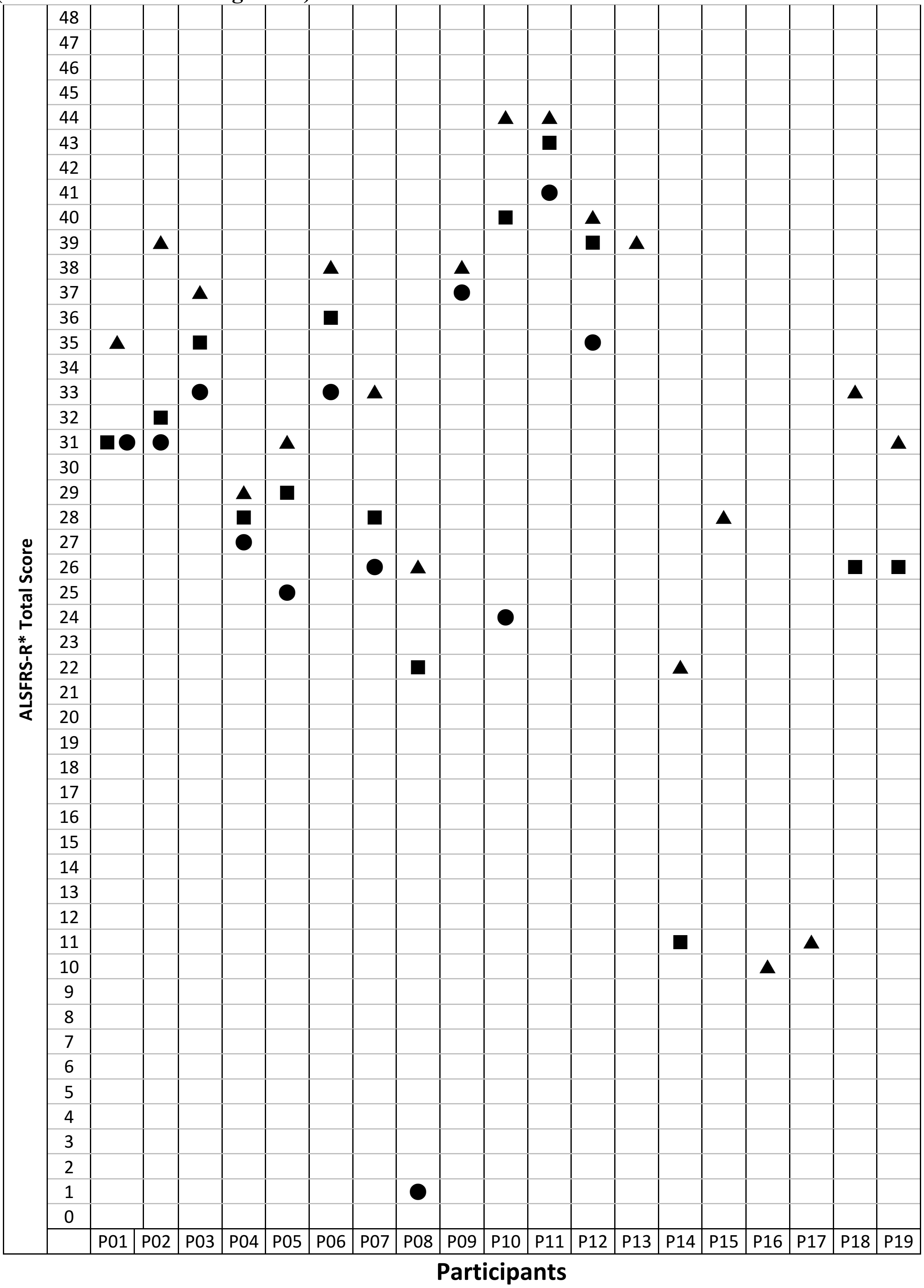

▲ Interview 1 (T1) ■ Interview 2 (T2) ● Interview 3 (T3)

*ALSFRS-R: ALS Functional Rating Scale (Cedarbaum et al., 1999)

Rating 48 = unimpaired function; Rating 0 = total dependence and reliance on invasive ventilation
